# The mortality risk after myocardial infraction in migrants compared with natives: a systematic review and meta-analysis

**DOI:** 10.3389/fcvm.2023.1101386

**Published:** 2023-05-24

**Authors:** Lei Zhu, Bao-tao Huang, Mao Chen

**Affiliations:** Department of Cardiology, West China Hospital, Sichuan University, Chengdu, China

**Keywords:** myocardial infraction, migrant, mortality, prognosis, meta analysis

## Abstract

**Background and Objective:**

The evidence on the risk of mortality after myocardial infarction (MI) among migrants compared with natives is mixed and limited. The aim of this study is to assess the mortality risk after MI in migrants compared to natives.

**Methods:**

This study protocol is registered with PROSPERO, number CRD42022350876. We searched the Medline and Embase databases, without time and language constraints, for cohort studies that reported the risk of mortality after MI in migrants compared to natives. The migration status is confirmed by country of birth, both migrants and natives are general terms and are not restricted to a particular country or area of destination or origin. Two reviewers separately screened searched studies according to selection criteria, extracted data, and assessed data quality using the Newcastle-Ottawa Scale (NOS) and risk of bias of included studies. Pooled estimates of adjusted and unadjusted mortality after MI were calculated separately using a random-effects model, and subgroup analysis was performed by region of origin and follow-up time.

**Result:**

A total of 6 studies were enrolled, including 34,835 migrants and 284,629 natives. The pooled adjusted all-cause mortality of migrants after MI was higher than that of natives (*OR*, 1.24; 95% *CI*, 1.10–1.39; *I*^2^*^ ^*= 83.1%), while the the pooled unadjusted mortality of migrants after MI was not significantly different from that of natives (*OR*, 1.11; 95% *CI*, 0.69–1.79; *I*^2^*^ ^*= 99.3%). In subgroup analyses, adjusted 5–10 years mortality (3 studies) was higher in the migrant population (*OR*, 1.27; 95% *CI*, 1.12–1.45; *I*^2^*^ ^*= 86.8%), while adjusted 30 days (4 studies) and 1–3 years (3 studies) mortality were not significantly different between the two groups. Migrants from Europe (4 studies) (*OR*, 1.34; 95% *CI*, 1.16–1.55; *I*^2^*^ ^*= 39%), Africa (3 studies) (*OR*, 1.50; 95% *CI*, 01.31–1.72; *I^2 ^*= 0%), and Latin America (2 studies) (*OR*, 1.44; 95% *CI*, 1.30–1.60; *I*^2^*^ ^*= 0%) had significantly higher rates of post-MI mortality than natives, with the exception of migrants of Asian origin (4 studies) (*OR*, 1.20; 95% *CI*, 0.99–1.46; *I*^2^*^ ^*= 72.7%).

**Conclusions:**

Migrants tend to have lower socioeconomic status, greater psychological stress, less social support, limited access to health care resources, etc., therefore, face a higher risk of mortality after MI in the long term compared to natives. Further research is needed to confirm our conclusions, and more attention should be paid to the cardiovascular health of migrants.

**Systematic Review Registration:**

https://www.crd.york.ac.uk/prospero/, identifier: r CRD42022350876.

## Introduction

Although the treatment and prognosis of myocardial infarction (MI) have improved greatly in recent decades, ischemic heart disease (IHD) was estimated to be responsible for more than 5 million deaths in 2019 and remains one of the leading causes of mortality in the world, bringing great burdens both to society and individuals (1). It is well known that the morbidity and mortality of cardiovascular diseases between different countries or regions are substantially different, such as the global burden of disease (GBD) study showed the incidence and prevalence of IHD in some high-income countries are higher than those in some low- and middle-income countries, although the disability adjusted life years (DALY) of IHD in some high-income countries are lower than those in some middle-income countries, and the age-standardized DALYs due to IHD were highest in Eastern Europe, Central Asia, Oceania, and the Middle East/North Africa, and relatively low in the rest regions of the world (1–3).

At the same time, with the continuous process of globalization, the number of international migrations has continued to grow in the past few decades. It is estimated that the number of persons living outside of their country of origin reached 281 million in 2020, among them, over 90 percent lived in high-income or upper middle-income countries, and about 76 percent came from a middle-income or low-income country (4). Nearly one in every six persons residing in a high-income country is a migrant, and they have profoundly changed the demographic structure of the destination country (4, 5). Therefore, the health status of the migrant population is of widespread concern.

Most studies have focused on the morbidity or mortality of diseases in the entire population of migrants compared to natives, including cardiovascular diseases, with mixed results (6). Some studies have observed that migrants are generally healthier than the natives, particularly in the early stages of migration, proposing the healthy migration hypothesis, whereby relatively healthier groups are more likely to migrate, and the salmon bias hypothesis, whereby less healthy migrants tend to return to their place of origin (7–9). These hypotheses and supporting observational data contradict the conventional view of migrants' health, as many studies also show that migrants often face many disadvantages, such as low socioeconomic status, language, cultural barriers, psychosocial stress, limited access to health care services, and therefore have poorer health (10, 11). A recent meta-analysis concluded that migrants had an advantage in mortality for most diseases, but no difference in ischemic circulatory disease; however, this study excluded certain patient groups, such as those already in the intensive care unit with MI (12). Despite MI is one of the main diseases that affect the human life span and there are large number of migrants with MI, studies investigating the outcomes of migrants with MI are limited.

The purpose of our study was to conduct a systematic review and meta-analysis to assess the prognostic differences between migrants and natives with MI and to explore the impact of migration on the prognosis of the migrant population with MI. The findings may help improve cardiovascular health in this large population and reduce health disparities while providing a specific perspective to further illustrate the mechanisms by which immigration affects health.

## Methods

This is a systematic review and meta-analysis that followed the PRISMA guidelines (13), and the protocol was registered with the prospective register of systematic reviews (PROSPERO; registration number CRD 42022350876). The PRISMA checklist is provided in the Supplementary Appendix S1.

### Eligibility criteria

We initially included studies that assessed the prognosis of migrant populations with MI. The study subjects were migrants with MI, whereas the comparison subjects were natives of the destination country with MI, and the main outcome was all-cause mortality after MI. MI are confirmed by ICD codes or clinical definition, and migrants are defined as those born in countries other than the country in which they currently live and the study was conducted. Both migrants and natives are general terms and are not restricted to a particular country or area of destination or origin. Studies were excluded if they met any of the following criteria: an editorial; a conference abstract; studies observing ethnic background only, with no explicit definition of migration; studies focused on subpopulations such as refugees; and a lack of a local resident control group.

### Search strategy

We searched MEDLINE via Ovid (from 1946 to November Week 1 2022) and EMBASE via Ovid (from 1974 to 2022 November 15) for published peer-reviewed literature with no restrictions on language and date. Additional records were identified by manual searches for references in the included studies. Using a Boolean search strategy developed by consulting previous literature (14, 15), detailed search terms are listed in Supplementary Appendix S2.

### Study selection

All citations in the search were downloaded to Endnote™^,^ and duplicate citations were removed. Two reviewers (LZ and BTH) independently screened the titles and abstracts of all citations in the database, and disagreements were resolved by consensus or by reference to the third reviewer (MC). The full texts of the included articles were assessed for eligibility by two reviewers independently using a checklist of inclusion and exclusion criteria, with reasons for exclusion noted, and disagreements were discussed by all authors to seek consensus.

### Data extraction

Two reviewers (LZ and BTH) independently collected data from the included studies: name of first author, year of publication, country, study design, country of origin, year of study, number of participants, age range, sex, number of mortality events, mortality rates, and follow-up time. Duplicate data were removed for studies reporting the same migrant group (by country of destination) for the same mortality outcome and period. Contradictions were resolved by consensus. Microsoft Office Excel was used to manage data extraction.

### Quality assessment

We used the Newcastle‒Ottawa Scale (NOS) to assess the risk of bias and methodological quality of the included studies (16). Studies scoring 0 to 3 points, 4 to 6 points, and 7 to 9 points were classified as low-, moderate- and high-quality studies, respectively. All studies were independently evaluated by two authors (LZ and BTH), and discrepancies were resolved by consensus or consultation with the third investigator (MC).

### Data analysis

The outcomes were the mortality rate after myocardial infarction, according to the follow-up time, which can be divided into ≤30 days mortality, 1 to 3 years mortality, and 5 to 10 years mortality. There are also several effect sizes with different adjustment factors in the same study, and we chose the one with the most adjustment factors. The heterogeneity among studies was assessed using *I^2^* statistics (17). Effect sizes were aggregated using fixed-effects or random-effects models according to the level of heterogeneity. Pooled estimates of odds ratios (ORs) with 95% confidence intervals were calculated to compare the unadjusted risk of mortality in migrants with natives, while pooled adjusted estimates of hazard ratios (HRs) with 95% confidence intervals were calculated to look at the adjusted risk of mortality in migrants with natives. Subgroup analysis was performed according to the region of the country of origin and the follow-up time, and all included mortality data were adjusted. The *p* value of statistical significance was set at 0.05. When *I^2^* exceeded 50%, we considered heterogeneity significant, and a sensitivity analysis was performed to explore potential sources of heterogeneity, including country of birth and follow-up time. Publication bias was assessed using the Egger test. All analyses were performed in STATA software 17.0 (Stata Corporation, College Station, TX, USA).

## Results

**Figure 1:**
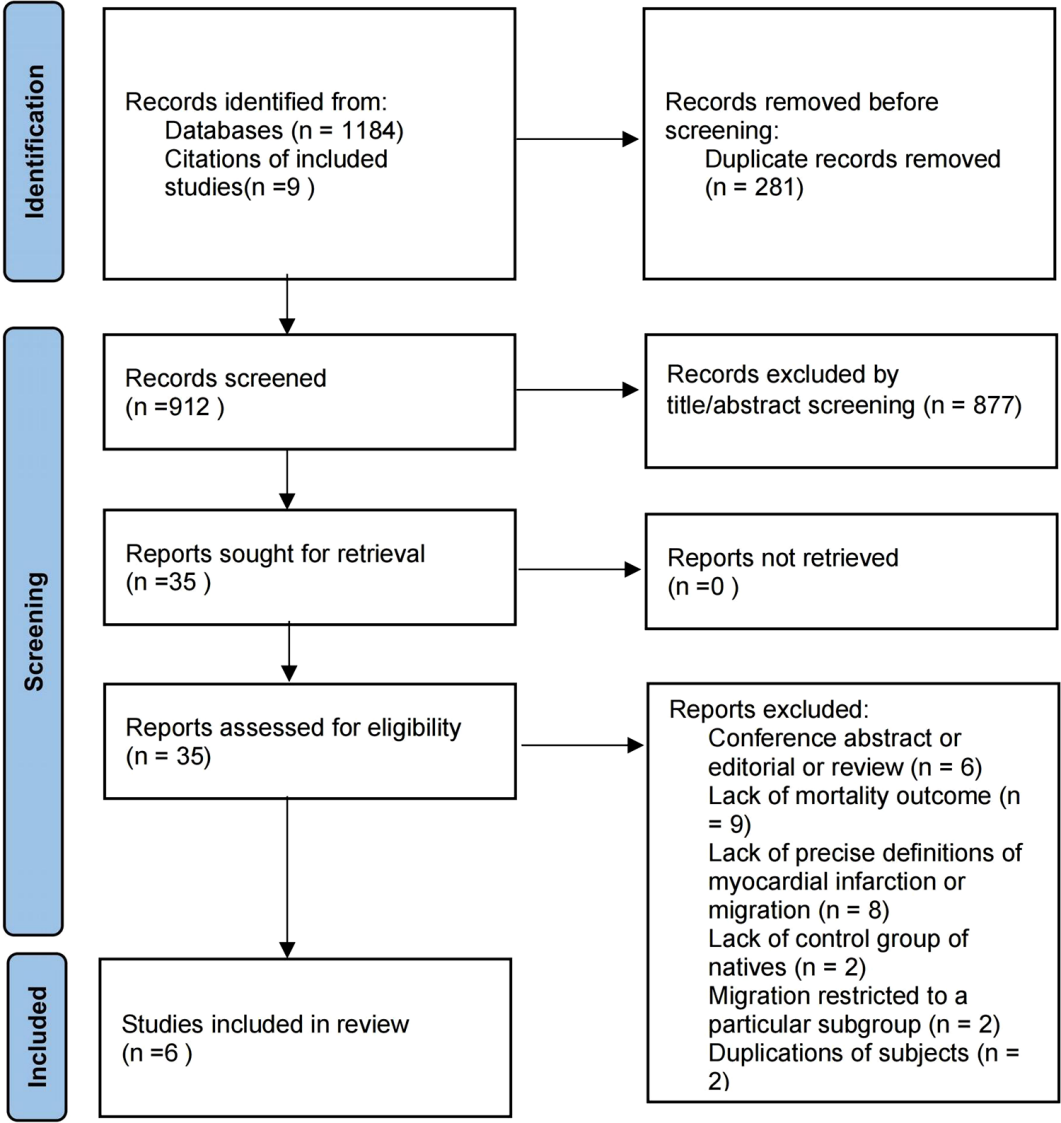
Flowchart of the study selection process.

We identified 1,184 publications through the literature search and screened them, 281 of which were duplicates. A total of 903 articles and an additional 9 articles identified by manual searches for references of included studies were screened by title and abstract, of which 35 were included in full-text screening, and 6 studies finally met the inclusion criteria (18–23). The reasons for exclusion are classified and given in Figure 1.

### Study characteristics and quality assessment

All included studies were conducted in high-income countries, including Israel (two studies), Sweden (two studies), The Netherlands, and Australia. These studies were published between 5 June 1997 and 22 August 2018, and included a total of 34,835 migrants (weighted average age is about 66.6 years old, and 69.7% male) and 284,629 natives (weighted average age is about 69.1 years old, and 65.4% male) with a recruitment period between 1981 and 2012, and migrants are mainly from Europe and Asia, and the weighted average follow-up time is about 8.1 years. The characteristics of the included studies are shown in Table 1. Using NOS to assess the quality of the 6 included studies, five studies (19–23) received a high-quality rating, and one study (18) received a moderate-quality rating. Overall, the qualities of the included studies were good. The detailed results table can be found in Supplementary Appendix S3.

**Table 1:** Characteristics of the included studies.

Source	Country of natives	Number of origin country or area	Number of Migrants	Age of Migrants	Male (%)	Number of natives	Age of Natives	Male (%)	Design and recruitment time	Definition of myocardial infraction	Length of follow-up time
Shvartsur R 2018 (19)	Israel	7	9,338	70.2 ± 12.6^a^	64	1,805	52.7 ± 11.3^a^	85.7	retrospective cohort study, 2002-2012	ICD-9-CM codes	10 years
Hedlund E 2008 (22)	Sweden	9	4,252	60.5^b^	82.7	20265	64.1^b^	72.5	retrospective cohort study, 1985-1996	ICD-8 codes	1 year
van Oeffelen AA 2014 (20)	The Netherlands	7	10,794	younger	72.1	2,02,836	69 (58–78)	66	retrospective cohort study, 1998–2010	ICD-9 codes	5 years
Rye E 2019 (18)	Australia	6	1,035	61 (52–70)^c^	82.1	1,087	58 (50–68)^c^	76.5	retrospective cohort study, 2004–2012	Clinical definition	30 days
Harpaz D 1997 (23)	Israel	6	5,059	64.4^b^	72.5	455	53.8^b^	84	retrospective cohort study, 1981–1983	Clinical definition	10 to 12 years
Yang D 2012 (21)	Sweden	6	4,357	67.8^b^	57	58,181	72.9^b^	60	retrospective cohort study, 1987–2008	ICD codes	21 years

^a^ means (SDs).

^b^ means.

^c^ medians (25th, 75th percentiles).

ICD-9-CM, The International Classification of Diseases, 9th Revision, Clinical Modification; ICD, The International Classification of Diseases.

## Main results

**Figure 2:**
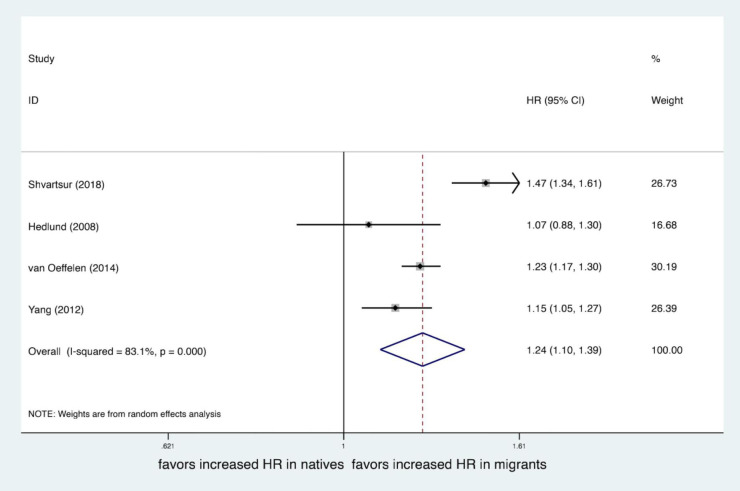
Forest plot of the adjusted hazard ratio (HR) of mortality after MI of migrants compared to natives.

**Figure 3:**
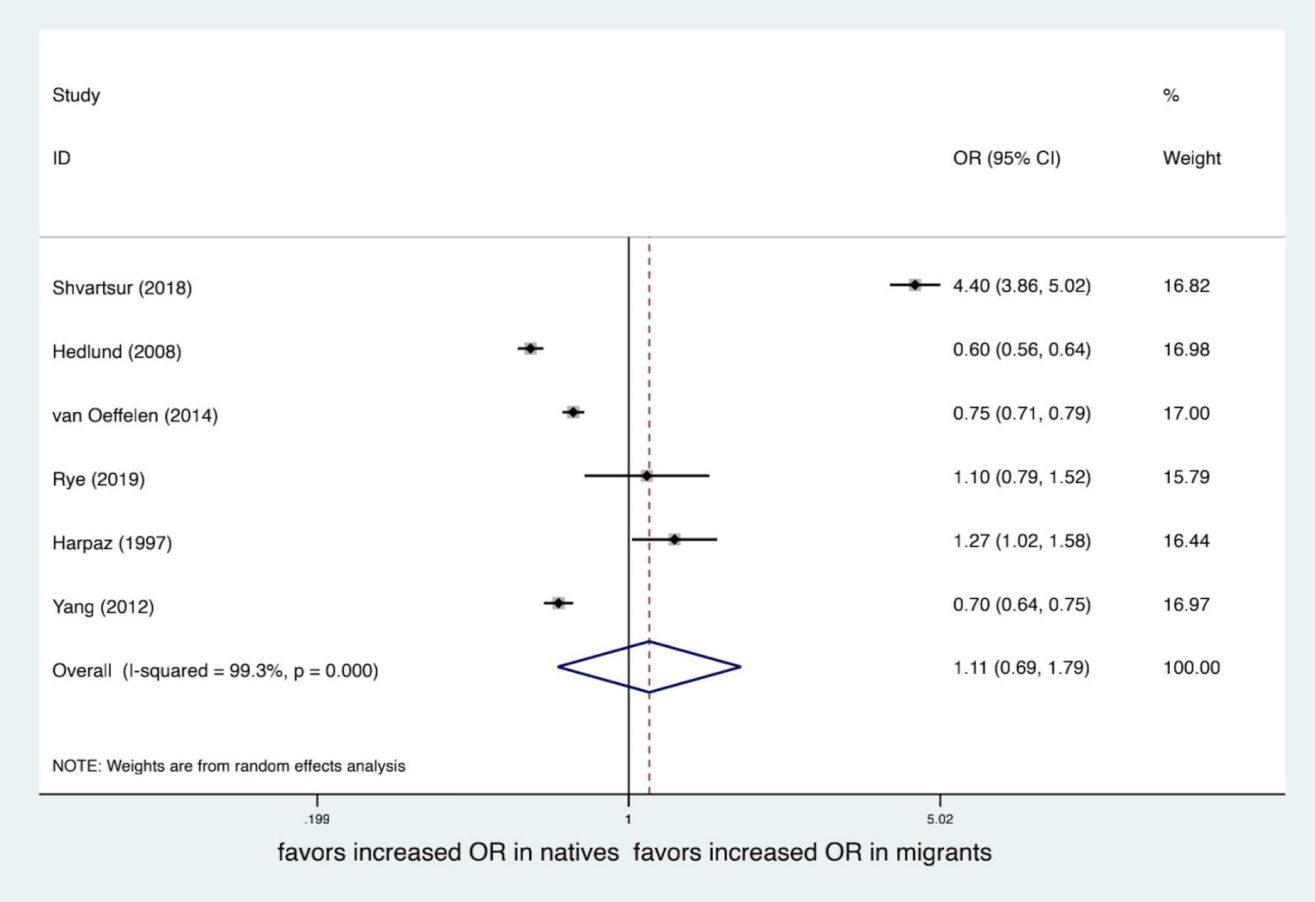
Forest plot of the unadjusted odds ratio (OR) of mortality after MI of migrants compared to natives.

Four studies (19, 20, 22, 23) provided an adjusted mortality rate, all including the adjustment factor of age, and the rest were gender, comorbidities, socioeconomic status, onset characteristics, year of event, etc. Mortality endpoint data with the longest follow-up time were selected in each study, and all mortality endpoints used in the adjusted analysis were followed up for more than 1 year. The pooled result showed that migrants had higher mortality after MI than natives (*OR*, 1.24; 95% *CI*, 1.10–1.39; *I*^2^*^ ^*= 83.1%), see Figure 2. While unadjusted mortality data were extracted from all the 6 studies, the pooled result showed that there were no statistically significant differences in mortality after MI among migrants compared to natives (*OR*, 1.11; 95% *CI*, 0.69–1.79; *I*^2^*^ ^*= 99.3%), see Figure 3.

### Sensitivity analyses

The heterogeneity between the studies was relatively high, so we used a random effect model and performed a sensitivity analysis. We conducted subgroup analysis by follow-up time and country of birth and found that the area of origin may explain part of the high heterogeneity, and planned subgroup analyses by reason f*or* migration, sex, and education were not practicable due to the lack of relevant data in most studies. For the meta-analysis of adjusted mortality, when the study by Shvartsur et al. was removed (19), heterogeneity was significantly lower (*OR*, 1.19; 95% *CI*, 1.11–1.27; *I*^2^*^ ^*= 31.7%), and the complex diversity of the included migrant populations and their origins may also contribute to the high heterogeneity (Supplementary Appendix S4).

## Results of the subgroup analysis

**Figure 4:**
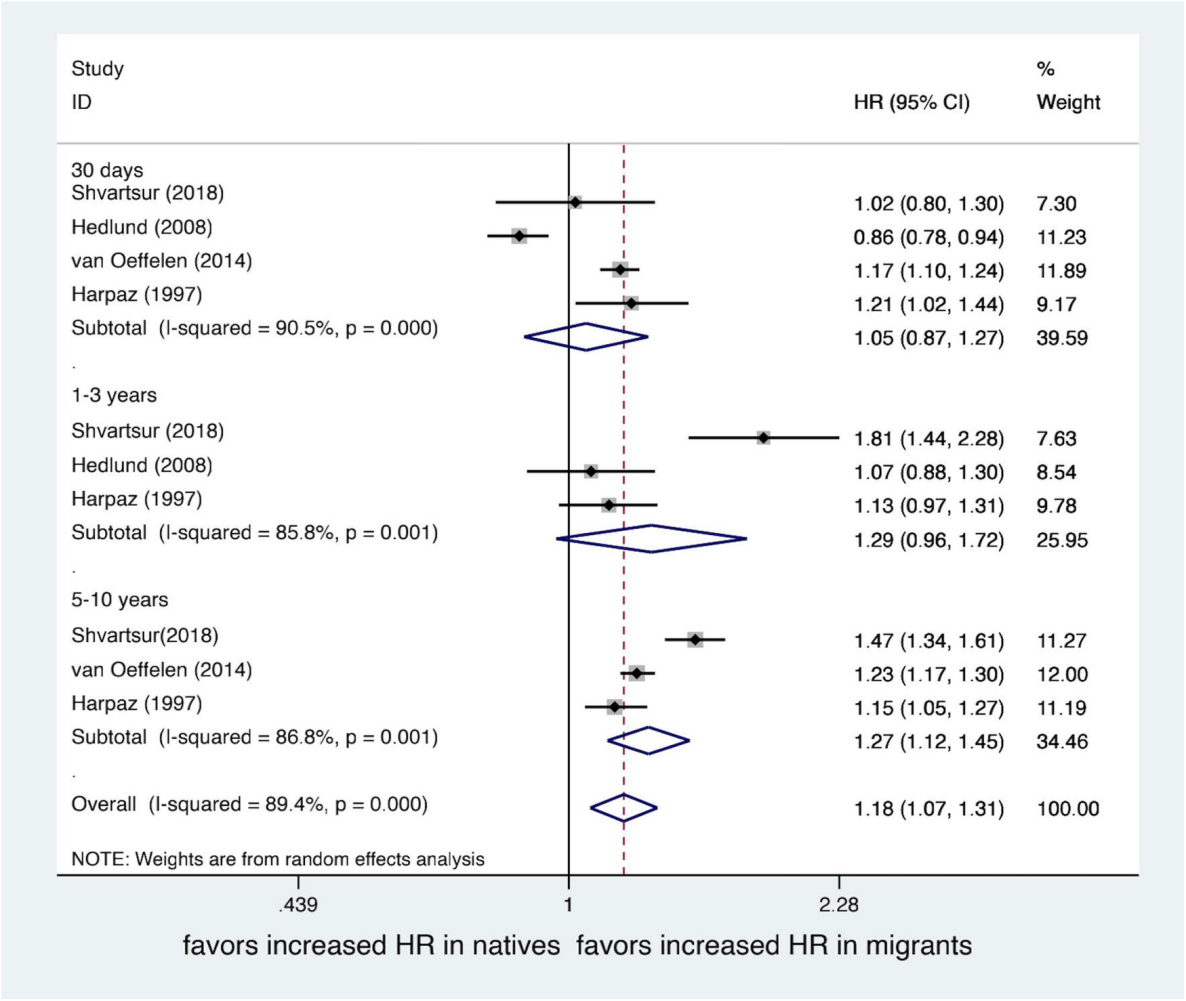
Forest plot of hazard ratio (HR) of mortality after MI of migrants compared with natives by follow-up time.

We performed a subgroup meta-analysis by the duration of follow-up. Four studies (19, 20, 22, 23) included in-hospital and 30-day mortality, 3 studies (19, 22, 23) included 1–3 years mortality, and 3 studies (19, 20, 23) included 5–10 years mortality. All included mortality data were adjusted. Pooled data showed 5–10 years mortality was significantly worse in migrants (*OR*, 1.27; 95% *CI*, 1.12–1.45; *I*^2^*^ ^*= 86.8%), while there is no statistically significant differences in 30 days mortality (*OR*, 1.05; 95% *CI*, 0.87–1.27; *I*^2^*^ ^*= 90.5%) and 1–3 years mortality (*OR*, 1.29; 95% *CI*, 0.96–1.72; *I*^2^*^ ^*= 85.8%) between migrants and natives. There was high heterogeneity between the groups. See Figure 4.

**Figure 5:**
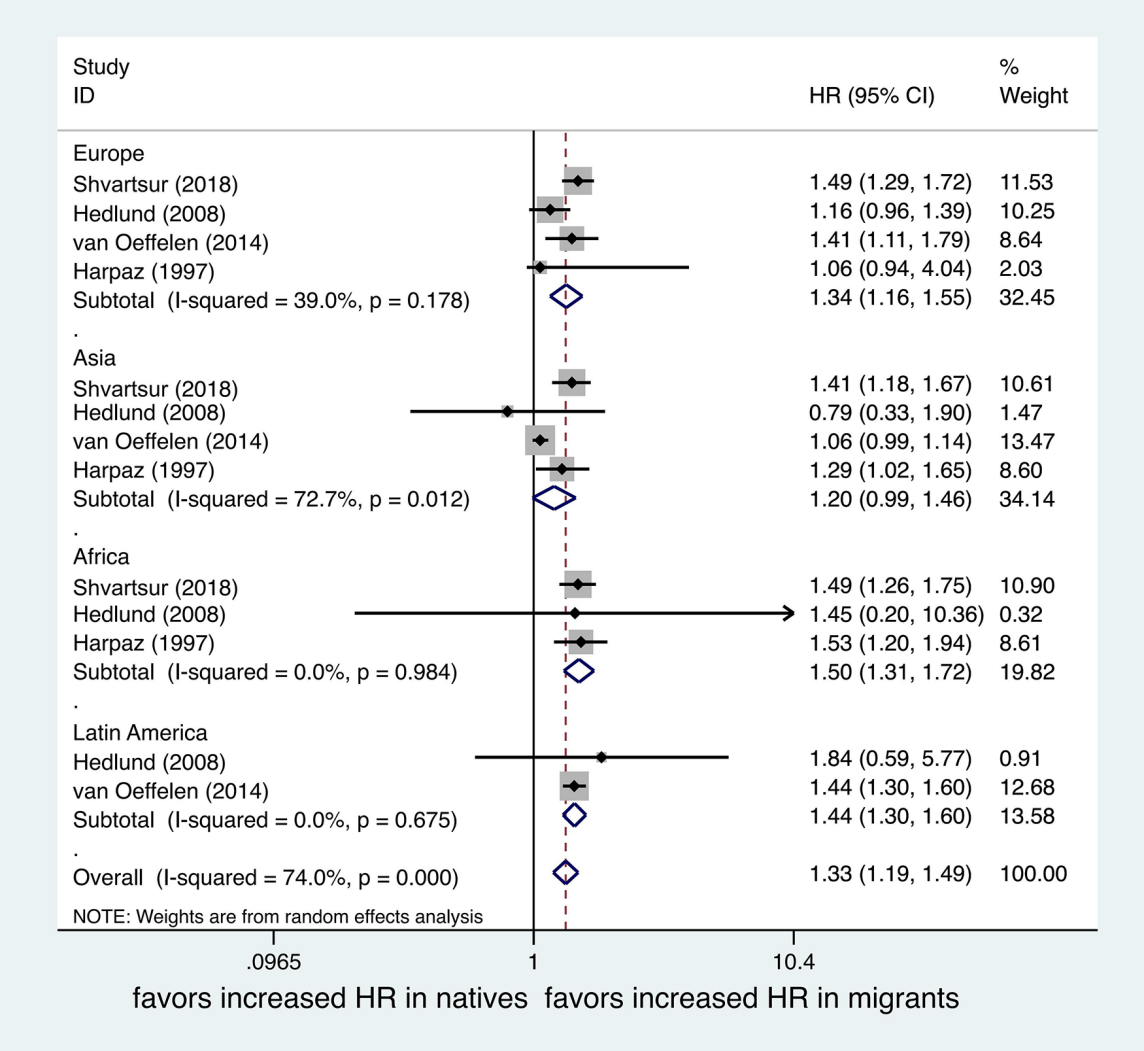
Forest plot of hazard ratio (HR) of mortality after MI of migrants compared with natives by area of birth.

A subgroup meta-analysis by area of birthplace was also performed. 4 studies (19, 20, 22, 23) included migrants of European and Asian origin, 3 studies (19, 22, 23) included migrants of African origin, and 2 studies (20, 22) included migrants of Latin American origin. All the endpoints of mortality were adjusted, and we also selected the one with the longest follow-up time. Migrants from Europe (*OR*, 1.34; 95% *CI*, 1.16–1.55; *I*^2^*^ ^*= 39%), Africa (*OR*, 1.50; 95% *CI*, 1.31–1.72; *I*^2^*^ ^*= 0%), and Latin America (*OR*, 1.44; 95% *CI*, 1.30–1.60; *I*^2^*^ ^*= 0%) had significantly higher rates of post-MI mortality than natives, with the exception of migrants of Asian origin (*OR*, 1.20; 95% *CI*, 0.99–1.46; *I*^2^*^ ^*= 72.7%). The heterogeneity between groups was small, except for the Asian subgroup. See Figure 5.

### Publication bias

Funnel plots, which are used to evaluate potential publishing biases, are usually displayed when more than ten studies are available and are not appropriate for meta-analysis which has a small number of eligible studies (24). Therefore, Egger's test was used and showed that there was no publication bias for the main unadjusted meta-analysis (Egger's test, *p *= 0.640 > 0.05) and the adjusted main meta-analysis (Egger's test, *p *= 0.870 > 0.05).

## Discussion

To our knowledge, this systematic review and meta-analysis is the most comprehensive and first to compare outcomes after MI between migrants and natives. The analysis concluded the following main results: compared to natives with MI, the long-term all-cause mortality (1–10 y) after MI of the migrants was higher, possible reason is the social-economic disadvantages migrant faces accumulate overtime; while the short-term all-cause mortality (≤30 d) was not, possibly because the high quality and homogeneous in-hospital treatment and insufficient follow-up time.

Many studies have compared cardiovascular outcomes between migrants and natives, but there is still no consensus. A review showed that cardiovascular outcomes among migrants vary depending on factors such as country of origin, country of destination and duration of migration (25). For example, in Spain, migrants from Asia, the Caribbean and sub-Saharan Africa had higher CVD mortality compared with natives, whereas migrants from North Africa and South America had lower CVD mortality (26). Genetic predisposition, lifestyle, exposure to risk factors, early life factors, socioeconomic factors, etc. that are related to the background of the migrants' country of origin have a major impact on the health status of migrants and are important causes of health disparities between migrants and the native population, as well as between migrants of different origins (11, 25, 26). We therefore conducted a subgroup analysis by migrants' country of origin, which reduces some of the heterogeneity.

There are also many other factors that may affect the cardiovascular health and prognosis of migrants, such as some studies have proposed the health migration effect hypothesis that immigrants tend to be healthier than the general population in their country of origin due to a selection process in which individuals with better health and socioeconomic status are more likely to migrate, and the salmon bias effect that immigrants who experience health problems or illnesses are more likely to return to their home country for medical care or to be with their families, while healthier immigrants tend to stay in the host country, leading to an overestimation of migrant health in the host country (7–9, 27, 28). However, many studies have also found that migrants tend to have a lower socioeconomic status (29–31), greater psychological stress (32, 33), less social support (11), and limited access to health care resources (34, 35), among other disadvantages, and therefore poorer health. For example, some studies have shown a higher prevalence and mortality of cardiovascular disease among migrants (26, 36). As MI is a common and serious cardiovascular disease and a major cause of cardiovascular death, it is important to study its status and prognosis in immigrant populations, and as mentioned above, factors such as socioeconomic status have an important influence on acute MI, but there is insufficient evidence on the prognosis of migrants compared with natives after MI, so we focus on migrant populations in whom MI developed (37, 38).

The pooled adjusted short-term mortality rate did not show significant differences between migrants and natives, and there are several possible reasons for this. The first may be due to the major advances in the treatment of MI, evidence shows that migrants do not differ from natives in terms of key in-hospital treatments, including rates of reperfusion therapy and door-to-balloon time, and no significant differences were observed in length of hospital stay, in-hospital complications, or mortality (18, 39–42). Although migrants are disadvantaged in terms of socioeconomic factors and psychological distress, high quality and homogeneous in-hospital care reduces these disparities, and the short follow-up period may not be sufficient to show a difference in the short-term prognosis of migrants compared to natives. On the other hand, the healthy migration effect observed in many studies from North America to Europe, supported by evidence ranging from physical health to mental health, suggests that the migrant group is a selected healthier group and may contribute positively to the prognosis of migrants, especially at the early stage (43–45). However, only patients who were admitted to the hospital were enrolled, ignoring those who did not present to the hospital after MI, and previous studies suggest that migrants may receive inferior pre-hospital care, which may underestimate short-term mortality of migrants (46, 47).

When the follow-up period was extended, the pooled data showed that the mortality rate of migrants was significantly higher than that of natives. Most migrants move to more developed areas, which also means that they tend to have poorer socioeconomic conditions and health awareness (11, 29–31, 48), and studies have shown that individuals with low socioeconomic status often bear a greater burden of CVD and are more likely to have a higher incidence of MI and poorer outcomes (37, 38). There are large number of studies have also shown that despite migrants receiving similar rates of reperfusion therapy as natives, there is often a delay in the time to treatment, with a study in Singapore showing significantly longer symptom-to-balloon times in migrant patients (40), another study in Australia showing that migrant patients spend longer time in emergency care (49), and several studies in the US showing that migrant patients spend longer in the time from symptom onset to door and door to balloon than natives (50, 51). At the same time, migrating to a new environment introduces barriers such as language and culture, which make migrant groups less likely to seek specialized medical care, less likely to communicate with doctors, and less likely to receive the standard of optimal drug treatment recommended by the guidelines and less likely to adhere to treatment (20, 52, 53). Furthermore, the advantages of the healthy migrant effect in migrants disappear over time, often 5–10 years after immigration, and the health status of migrants is equal to or worse than that of natives (7, 43, 54). For example, cultural adaptation leads to lifestyle changes, including changes in physical activity levels and diet, resulting in increased rates of hypertension, obesity, and diabetes in migrants (55), which are traditional risk factors for MI. Over time, the adverse effects of these disadvantages accumulate, leading to a poorer prognosis for migrants compared to natives.

## Strengths and limitations

This study aggregates existing data to complement evidence on the risk of mortality after MI in migrants compared to natives and draws interesting conclusions that inform future research and provide a basis for more targeted health policies. At the same time, the migrant groups included in this study cover a wide range of source regions and are representative to some extent. Furthermore, data from all patients were obtained from medical records, and the follow-up endpoint was all-cause mortality, so there is a high precision of the data.

There are some limitations to consider. First, all included studies were conducted in developed countries, and therefore, the conclusions are not representative of less developed countries and regions. Second, the follow-up endpoint is only death, and while improving precision, it lacks other important indicators, such as the occurrence of MACE (major adverse cardiovascular event) and quality of life; also, it cannot be ruled out that all-cause death may have been disturbed by confounding factors other than MI. Next, all studies included in the analysis were retrospective, and prospective studies in this area are needed in future research. Last, the heterogeneity between studies was very large, although the heterogeneity of subgroup analysis by region of origin was significantly reduced, more detailed data are lacking to further explore the source of heterogeneity. However, these limitations will not affect the need of strengthening our focus on the health of migrants.

## Conclusions

In this systematic review and meta-analysis, we summarize the existing research data and conclude that the mortality risk of migrants is higher than that of natives in the long term after MI. Given the large number of migrants and the significant burden of MI, this study is important and highlights the need to improve the cardiovascular health of migrants and reduce health inequities. More researches are needed to explore the differences between migrants and natives in more cardiovascular diseases and the underlying mechanisms, especially among migrants who already develop diseases.

## Data Availability

The original contributions presented in the study are included in the article/**Supplementary Material**, further inquiries can be directed to the corresponding author/s.
